# COVID-19 Community Transmission among Healthcare Workers at a Tertiary Care Cardiac Center

**DOI:** 10.3390/medsci9030049

**Published:** 2021-06-30

**Authors:** Mazin Barry, Asirvatham Alwin Robert, Mohamad-Hani Temsah, Syed Abdul Bari, Muhammad Yasin Akhtar, Faizah Al Nahdi, Richilda Erlandez, Jaffar A. Al-Tawfiq, Abdullah Al Khushail, Yahya Al Hebaishi

**Affiliations:** 1 Division of Infectious Diseases, Department of Internal Medicine, College of Medicine, King Saud University, Riyadh 11461, Saudi Arabia; 2 Department of Endocrinology and Diabetes, Prince Sultan Military Medical City, Riyadh 12233, Saudi Arabia; aalwinrobert@gmail.com; 3 Pediatric Department, College of Medicine, King Saud University, Riyadh 11461, Saudi Arabia; mtemsah@ksu.edu.sa; 4 Department of Infection Control, Prince Sultan Cardiac Center, Riyadh 12233, Saudi Arabia; drsabari7may@gmail.com (S.A.B.); makhtar@pscc.med.sa (M.Y.A.); f.alnahdi@pscc.med.sa (F.A.N.); rerlandez@pscc.med.sa (R.E.); 5 Specialty Internal Medicine and Quality Department, Johns Hopkins Aramco Healthcare, Dhahran 31311, Saudi Arabia; jaltawfi@yahoo.com; 6 Infectious Disease Division, Department of Medicine, Indiana University School of Medicine, Indianapolis, IN 46202, USA; 7 Infectious Disease Division, Department of Medicine, Johns Hopkins University School of Medicine, Baltimore, MD 21287, USA; 8 Department of Adult Cardiology, Prince Sultan Cardiac Center, Riyadh 12233, Saudi Arabia; aalkhushail@pscc.med.sa (A.A.K.); yalhebaishi@pscc.med.sa (Y.A.H.)

**Keywords:** healthcare workers, cardiac center, COVID-19, community transmission, mortality, Kingdom of Saudi Arabia

## Abstract

**Aim:** To determine the frequency, mode of transmission, and outcome of Coronavirus Disease 2019 (COVID-19) among healthcare workers (HCWs) in a tertiary care cardiac center in the Kingdom of Saudi Arabia (KSA). **Methods:** This is a retrospective study of Severe Acute Respiratory Syndrome Coronavirus 2 (SARS-CoV-2) infected HCWs and was conducted from 2 March to 31 December 2020. Data related to the presence of COVID-19 symptoms, mode of transmission, hospitalization, and mortality were collected from the patients’ medical records. **Results:** Of the 4462 patients tested for COVID-19 by real-time reverse transcriptase polymerase chain reaction (RT-PCR), 203 (4.5%) HCWs were positive; of these, 125 (61.6%) were males, and the most common age group was <40 years. The most commonly encountered health professionals were nurses (74, 36.4%), followed by therapists/technicians (48, 23.6%), housekeepers (25, 12.3%), and physicians (21, 10.4%). The majority (184, 90.6%) of the HCWs contracted COVID-19 in the community, and only 19 (9.4%) were healthcare-associated infections. Of the infected HCWs, 169 (83.3%) had mild symptoms and were managed in home isolation. The most common symptoms were fever (128, 63.1%), body ache (124, 61.8%), headache (113, 55.7%), dry cough (123, 60.6%), sore throat (97, 47.8%), body weakness (97, 47.8%), and fatigue (94, 46.3%). Comparing males and females, there was a significantly higher number of female nurses; in contrast, there was a higher number of male physicians, housekeepers, therapists/technicians, and other specialty HCWs. A significantly lower number of nurses, therapists/technicians were infected in the ≥40 years age group compared to <40 years. Furthermore, a significantly higher difference was observed among non-Saudi nurses compared to Saudi nurses. No mortality was documented among the included HCWs. **Conclusions:** In the largest tertiary cardiac center in KSA, most HCWs who contracted COVID-19 developed mild symptoms; nurses and those aged <40 years were most commonly infected, and most infections were acquired in the community. HCWs’ adherence to mitigation measures outside of the workplace is vital to curb the current pandemic and decrease nosocomial transmission risk.

## 1. Introduction

In the past two decades, there have been three major outbreaks of coronaviruses that have affected the global population, including the most recent pandemic of Coronavirus Disease 2019 (COVID-19). The disease was initially reported in China [1], and on 30 January 2020, the World Health Organization (WHO) declared the COVID-19 outbreak to be a Public Health Emergency of International Concern (PHEIC) [2]. In the Kingdom of Saudi Arabia (KSA), the government had taken several actions to control and lessen the pandemic impact and then allowed gradual returns to routine life [3,4].

In KSA, the majority of infected or symptomatic patients seek medical treatment in large healthcare facilities rather than clinics, and thus, any increase in the number of COVID-19 cases will impact the daily activity and capacity of these hospitals [5]. It is known that COVID-19 had overwhelmed hospitals and intensive care unit (ICU) admissions worldwide. Thus, it is critical to maintain preventive and curative services, especially for the most exposed people, such as healthcare workers (HCWs) [6,7]. HCWs may be unintentionally exposed to COVID-19 patients, and thus are at an increased risk of contracting the disease or even dying due to occupational exposure combined with long working hours, stress, and fatigue [8,9]. In an earlier study, the COVID-19 infection rate was higher among HCWs (4.1–38.9%) than in the general population (5.1–5.7%) [10].

The Saudi Ministry of Health (MOH) confirmed the first case of COVID-19 on 2 March 2020. At this time, it is challenging to forecast the final outcomes of the COVID-19 pandemic in KSA. As of 29 May 2021, KSA had reported 448,284 cases (out of 35,341,000 total populations) and 7334 confirmed deaths [11,12]. Several studies have recently been published regarding COVID-19 in KSA, but limited research has focused on HCWs. KSA has 498 hospitals with a large number of HCWs, and thus, it is essential to understand the occurrence and mode of transmission of COVID-19 among HCWs. Infection among HCWs may lead to the transmission of Severe Acute Respiratory Syndrome Coronavirus 2 (SARS-CoV-2) infection to vulnerable patients and may further deplete the workforce with further critical shortages of HCWs and adversely impact patient care [13,14].

Previous studies showed that patients with cardiovascular disease (CVD) are at an increased risk for developing severe COVID-19 symptoms, poor prognosis, and a high mortality rate, placing HCWs in a vulnerable position as they become hosts or vectors of viral transmission [15]. Studies in the Middle East region among HCWs in cardiac centers are lacking. Therefore, the present study aimed to determine the frequency, mode of transmission, and outcomes of COVID-19 among HCWs in a cardiac center in KSA.

## 2. Methods

### 2.1. Study Design and Setting

This study is a retrospective analysis of HCWs infected with SARS-CoV-2 and was conducted from 2 March 2020 to 31 December 2020 at Prince Sultan Cardiac Center (PSCC), Riyadh, KSA. PSCC is one of the leading cardiac centers (184 beds, 2331 employees) in the Middle East.

### 2.2. Ethical Approval

The study protocol was approved by the Research and Ethics Committee of PSCC, Riyadh, KSA (Ethical Approval No. 2020-027) and informed consent was obtained from all participants.

### 2.3. Measurements and Definitions

COVID-19 diagnosis was made following the interim guidance of the WHO. All suspected symptomatic and asymptomatic cases of COVID-19 and all their contacts as determined by contact tracing underwent nasopharyngeal swabs (NPS), and no periodic testing for all HCWs was done at the center during the study period. NPS were sent via viral transport media (Copan, Brescia, Italy). A confirmed COVID-19 case was defined as having a positive result for both SARS-CoV-2 E and S genes using a RealStar^®^ SARS-CoV-2 real-time reverse transcriptase polymerase chain reaction (RT-PCR) kit (Altona^®^-Diagnostics, Hamburg, Germany) and the Rotor-gene Q system (Qiagen^®^, Santa Clarita, CA, USA) in our hospital molecular laboratory.

### 2.4. Home Isolation

Patients who were asymptomatic and those who were clinically stable, with mild symptoms, no oxygen requirements, and no evidence of pneumonia, were placed on home isolation in accordance with the hospital infection control guidelines.

### 2.5. Hospital Admission

Patients who presented with any of the following features were admitted to the hospital: a respiratory rate of 30/min or more; blood oxygen saturation of 93% or less; partial pressure of oxygen/fraction of inspired oxygen ratio below 300; lung infiltrates above 50% of the lung field; acute respiratory distress syndrome, sepsis, altered consciousness, multi-organ failure, or cytokine release syndrome [16].

### 2.6. Contact Tracing

The infection control department defined a contact as anyone exposed to a confirmed COVID-19 case from two days before that case’s onset of symptoms (or positive PCR if asymptomatic) until 14 days after the last exposure to that case in any of the following settings: close contact within two meters for more than 15 min; direct physical contact; providing direct care without appropriate personal protective equipment; living in the same household; sharing a room, meal, or other space; sitting within two rows in any direction for more than 15 min, or any crew in direct contact with a case in public or shared transportation. Contacts were thus divided into either hospital or community-acquired infections.

### 2.7. Data Collection

The electronic medical records were reviewed for all identified HCWs with COVID-19 who were admitted to the hospital and those under home isolation. Information on patient age, gender, job category, and treatment location were gathered. Further data on the presence of COVID-19 symptoms, such as fever, dry cough, sore throat, body ache, loss of appetite, chills, fatigue, headache, vomiting, diarrhea, loss of taste, loss of smell, shortness of breath, runny nose, and body weakness, were also collected for all HCWs with COVID-19. Infection control practitioners at the hospital approached all positive HCWs (either home isolated or hospital admitted) through physical visits for interviews and follow-up daily telephone calls to gather data regarding exposure and other related histories as part of their contact tracing investigation and categorized staff accordingly to either hospital-acquired or community transmission based on contact tracing investigation.

### 2.8. Statistical Analysis

Data analysis was carried out using Microsoft Excel 2010 (Microsoft Corporation, Seattle, WA, USA) and IBM SPSS Statistics for Windows, version 22 (IBM Corp., Armonk, NY, USA). The continuous variables are represented as mean ± SD, while the categorical variables are shown as frequencies and percentages. Chi-squared tests (for categorical variables) were also performed to determine statistical significance. A *p*-value of less than 0.05 was considered statistically significant.

## 3. Results

**Figure 1:**
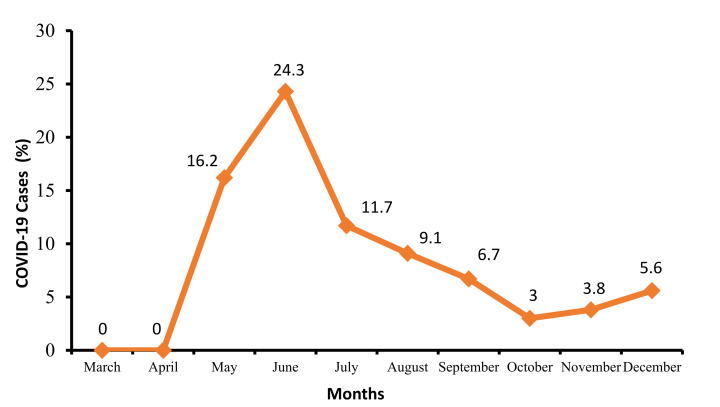
Epidemiological curve of the monthly rate of COVID-19 cases at Prince Sultan Cardiac Center in 2020.

During the study period, 4462 patients (2331 HCWs and 2131 non-HCWs) underwent SARS-CoV-2 RT-PCR testing, and 358 (8.0%) were positive. Of the positive cases, 155 (43.3%) were non-HCWs, 203 (56.7%) were HCWs, comprising 4.5% of the total tested positive. From a total of 2331 HCWs within the center, the prevalence was 8.7%. The monthly numbers of COVID-19 cases at PSCC are shown in Table 1 and Figure 1. The highest rate of positive HCWs was in the month of June 2020 with 24.3%.

Demographic characteristics of the study sample of 203 HCWs are shown in Table 2. Of the HCWs, 125 (61.6%) were males. The mean age was 37.3 ± 9.1 (range 21–62) and the most common age group was 30–39 years.

Infection rate of the total HCWs working in the hospital in each category was for nurses: 74 out of 638 (11.6%), physicians: 21 out of 208 (10.1%), therapists/technicians: 48 out of 366 (13.1%), and for housekeepers: 25 out of 146 (17.1%).

**Figure 2:**
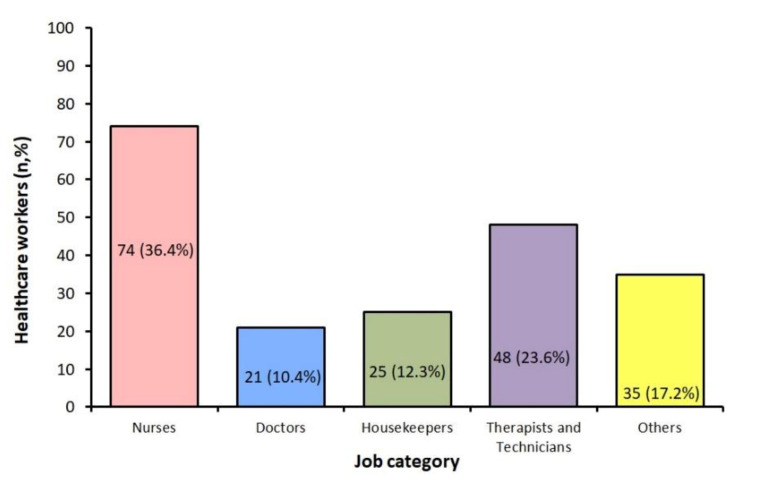
Number of COVID-19 cases among healthcare workers by job category.

Figure 2 shows COVID-19 among the 203 HCWs in relation to the job category. The most commonly affected groups were nurses (36.4%), followed by therapists/technicians (23.6%), housekeepers (12.3%), and physicians (10.4%).

Table 3 presents the most commonly reported symptoms. The most common symptoms were fever (128, 63.1%), body aches (124, 61.8%), headache (113, 55.7%), dry cough (123, 60.6%), sore throat (97, 47.8%), body weakness (97, 47.8%), and fatigue (94, 46.3%).

**Figure 3:**
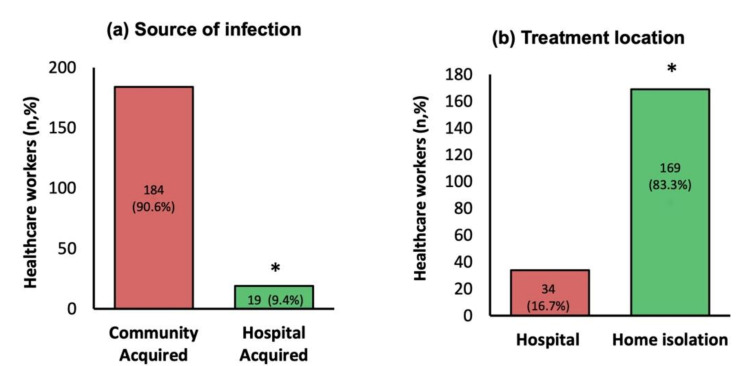
(**a**) Source of infection as determined by infection control contact tracing investigation, (**b**) treatment location of HCWs. Groups compared using the chi-squared (nonparametric) test, * *p*-values of <0.05 are considered statistically significant.

Figure 3a displays the source of infection as per infection control department contact tracing.

The majority of the HCWs (184, 90.6%) acquired COVID-19 in the community—84 (41%) from roommates or social gatherings with other HCWs, 55 (27%) from family members, 32 (16%) from shared transportation with HCWs, 30 (15%) from undetermined community transmission, and 2 (1%) as returning travelers. Only 19 (9.4%) HCWs had healthcare-associated infections of SARS-CoV-2. Of the infected HCWs, 169 (83.3%) were asymptomatic or had only mild symptoms and were managed under home isolation conditions, while 34 (16.7%) required hospitalization (Figure 3b).

Table 4 shows the results of gender, age, nationality, and treatment location comparisons. Compared to males, a significantly higher number of female nurses were infected; in contrast to the nurse group, a lower number of female doctors, housekeepers, therapists/technicians, and other specialty HCWs were infected. Regarding the age group, a significantly lower number of nurses, therapists/technicians were infected in the ≥40 years age group compared to <40 years. Furthermore, a significantly higher difference was observed among non-Saudi nurses compared to Saudi nurses. Significantly, a higher number of nurses, doctors, therapists/technicians, and other specialty HCWs were treated at home except for 96% of housekeepers treated at the hospital. There were no re-infections during the study period, and no mortality was documented among the included HCWs.

## 4. Discussion

The COVID-19 pandemic has overwhelmed hospitals in many countries and had imposed extra-burden on the HCWs [17]. Since the beginning of the pandemic, the healthcare community has gained valuable clinical experience in providing care for COVID-19 patients [18]. Several studies have also been conducted in KSA, but few have been conducted among HCWs [19,20,21]. This study, therefore, sought to explore COVID-19 infections among HCWs in KSA.

In the present study, as per the infection control department contact tracing investigation, the majority of infected HCWs (90.6%) contracted SARS-CoV-2 infection in the community, while only 9.4% acquired the infection in the hospital. An earlier report from KSA showed that 88% of infections in HCWs were hospital-acquired, mostly during a single hospital outbreak [21]. It should be noted that PSCC did not witness any hospital outbreaks. In addition, most community infections were related to housing conditions, because non-Saudi HCWs, including nurses and housekeepers, live in compounds with shared rooms and facilities. It had been suggested that there is a need to develop suitable accommodation and to have designated housing for exposed HCWs [22]. These types of accommodations were reported to play a role in outbreaks of the Middle East respiratory syndrome coronavirus (MERS-CoV) in KSA [23]. Recent reports have confirmed that most HCWs acquire infection in the community and have emphasized the importance of risk mitigation outside the workplace [24]. A study from Oman (east border of Saudi Arabia) showed 61.3% of HCWs acquired SARS-CoV-2 infection in the community compared to 25.5% of hospital-acquired [25]. Another study showed that 6.8% of 2842 COVID-19 patients were HCWs [26]. HCWs compliance rate to personal protective equipment (PPE) was consistent throughout the study period; however, the peak of the cases among HCWs was in June 2020. This peak correlates with the increased number of COVID-19 in all the regions of KSA at that time period, as reported previously [27].

The largest group of infected HCWs were nurses. One of the key reasons that personnel in nursing-related professions are at higher risk is their regular and close contact with patients, leading to a longer cumulative exposure time. The US Centers for Disease Control and Prevention (CDC) reported that HCWs who developed COVID-19 had a longer exposure time to the index patient [28]. Furthermore, nursing-related professions account for a large proportion of the healthcare workforce. Another study explored hospitalization data from 13 sites in the United States and found that 6% of the adults hospitalized were HCWs, of whom 36% were in nursing-related occupations, which is similar to the current study findings [29]. Studies have also reported that nurses are on the frontline and responsible for providing holistic care for all patients, and therefore have a higher probability of acquiring COVID-19 than other HCWs [30]. In a report, the International Council of Nurses confirmed that nearly 1500 nurses have died from COVID-19 in 44 countries and estimated that COVID-19 fatalities among HCWs worldwide could exceed 20,000 [31]. Knowing that those working in hospitals are at higher risk of secondary infection or spreading the virus to colleagues, family, and friends, particular attention should be drawn to proper protection and infection control measures for nurses to prevent further spread [32].

Our study found 19 (25.7%) Saudi nurses infected with COVID-19 compared to 55 (74.3%) non-Saudi nurses. An earlier study found that non-Saudi nurses self-reported greater awareness, better prevention, and better attitudes toward COVID-19 than Saudi nurses [33]. This is to be expected, as frontline nurses are likely to be better prepared, and most of the frontline nurses in the country are expatriates [34]. Fortunately, the majority (72, 97.3%) of the nurses had only mild infections and were treated at home. The present study also showed that more non-Saudi physicians (15, 71.4%) than Saudi physicians (6, 28.6%) were diagnosed with COVID-19, but all infected physicians similarly had mild infections and were treated at home.

It is important to note that, overall, the Middle East and North Africa, except Iran, have experienced much lower rates of COVID-19 than Europe and the United States [35]. The region has previously witnessed outbreaks of other novel coronaviruses, including MERS-CoV, and the H1N1 influenza pandemic. The affected countries consequently improved their infection control measures, and many have been proactive in responding to COVID-19 by initiating mitigation efforts to contain the infection before identifying their first case [36,37,38,39].

Importantly, there were no fatalities among the 203 infected HCWs and only 34 required hospitalizations. A previous report in KSA also reported no mortality among infected HCWs [21]; these favorable outcomes are interesting and need to be further validated by nation-wide surveys of infected HCWs.

According to the WHO, Middle East countries have seen fewer deaths, with a 1.8% mortality rate compared to the global average of 3.36% [35]. According to a recent wide-ranging data analysis by Hong Kong-based Deep Knowledge Group, KSA has been among the world’s 20 safest nations during the COVID-19 pandemic [40]. Similarly, these lower-case fatality rates need further exploration in future prospective studies.

The present study shows male predominance among infected HCWs (61.6% vs. 38.4%); this may be due to the fact that all housekeepers and a higher number of therapists/technicians in the studied population are male. A study from Qatar (border country of Saudi Arabia) showed a higher number of male HCWs (65.6%) were affected by COVID-19 [41]. However, these findings are different from other studies that reported that female HCWs are affected in higher numbers [26]. A study showed that 75.5% of 49 HCWs with COVID-19 were female [42]. These differences might reflect the different workforce constitutions of any given organization, although many studies show that males and females have the same prevalence of COVID-19, with male patients having higher risk for worse outcomes and death [43,44].

We found that the higher infected age category was <40 years in the present study. An earlier study conducted across all KSA regions during April and May 2020 also reported that the 30–39-year age group had the highest risk of contracting COVID-19, explaining it to be expected in KSA as the majority of workers are within this age group [45], whereas another possible explanation extrapolated from our findings is that this specific age group tend to be more commonly living in shared rooms. Our study also showed that non-Saudis were infected in higher numbers than Saudis, which is similar to previous findings [45], and close-quarter living might be the reason for this, as non-Saudis are the ones living in shared apartments, often with multiple roommates with shared bathrooms and dining areas. Future research analyzing infection rates in such accommodations for HCWs are warranted.

In this study, we found that some of the most common symptoms were fever (63.1%), cough (60.6%), and sore throat (47.8%). An earlier study from KSA stated that the most common symptoms were cough (89.4%), fever (85.6%), and sore throat (81.6%) [19]. The present study found that gender, age, nationality, and treatment location showed significant differences in case numbers, which agrees with earlier findings from KSA [23,45]. However, a limited number of infected HCWs were above the age of 65, which is associated with worse outcomes [46]. There were no fatalities among this cohort, as noted previously [26].

The limitations of this study comprise the relatively small sample size; the limited number of risk factors and comorbidities examined; the limited social and demographic factors examined; a lack of data on symptom duration; the absence of cycle threshold (Ct) values for PCR and follow-up negative PCR results; and restriction to a single cardiac center. Although the limitations exist in the present investigation, the present study delivers valuable data about the existing risk of COVID-19 among HCWs and provides helpful modifiable insights.

## 5. Conclusions

In the largest tertiary care cardiac center in KSA, nurses and the <40 years age group were the highest infected HCWs. Most HCWs developed mild COVID-19 symptoms, there was no mortalities, and most infections were community-acquired. This calls for future actions by enhancing COVID-19 prevention and control and further research studying COVID-19 community transmission among HCWs and its impact on frontline healthcare providers.

## Figures and Tables

**Table 1:** Rate of monthly COVID-19 cases at Prince Sultan Cardiac Center from 2 March to 31 December 2020.

Month	Number of Patients Screened with PCR for SARS-CoV-2	Number SARS-CoV-2 Positive Tests		
		Healthcare Workers n (%)	Patients n (%)	Total n (%)
March	7	0 (0)	0 (0)	0 (0)
April	43	0 (0)	0 (0)	0 (0)
May	111	14 (12.6)	4 (3.6)	18 (16.2)
June	403	74 (18.4)	24 (5.9)	98 (24.3)
July	452	36 (7.9)	17 (3.8)	53 (11.7)
August	639	25 (3.9)	33 (5.2)	58 (9.1)
September	715	18 (2.5)	30 (4.2)	48 (6.7)
October	769	7 (0.9)	16 (2.1)	23 (3)
November	808	15 (1.9)	16 (1.9)	31 (3.8)
December	515	14 (2.7)	15 (2.9)	29 (5.6)
Total	4462	203 (4.5)	155 (3.5)	358 (8.0)

PCR: polymerase chain reaction, COVID-19: coronavirus disease—2019, SARS-CoV-2: severe acute respiratory syndrome coronavirus 2.

**Table 2:** Demographic characteristics of the study sample.

Demographic Variables	Frequency (n = 203)	Percentage
**Gender**		
Male	125	61.6
Female	78	38.4
**Age**		
<40 years	130	64
≥40 years	73	36
**Mean Age**	37.3 ± 9.1 (range 21–62)	
**Nationality**		
Saudi	75	36.9
Filipino	49	24.1
Indian	35	17.2
Malaysian	17	8.4
Pakistan	11	5.4
Sudanese	5	2.5
Egyptian	4	2
Bangladesh	2	1
Palestinian	2	1
South African	1	0.5
Tunisian	1	0.5
Yemeni	1	0.5
**Travel History Overseas**		
Yes	2	1
No	201	99
**Mortality**		
Yes	0	0
No	203	100

**Table 3:** COVID-19 symptoms among all healthcare workers.

Symptoms	Frequency (n = 203)	Percentage
Fever	128	63.1
Dry cough	123	60.6
Sore throat	97	47.8
Body aches	124	61.8
Loss of appetite	73	36
Chills	57	28.1
Fatigue	94	46.3
Headache	113	55.7
Vomiting	18	8.9
Diarrhea	41	20.2
Loss of taste	76	37.4
Loss of smell	73	36
Shortness of breath	50	24.6
Runny nose	65	32
Body weakness	97	47.8
Asymptomatic	6	2.9

**Table 4:** Gender, age, nationality, and treatment location comparisons among healthcare workers.

	Total N = 203 n (%)	Nurses N = 74 n (%)	Doctors N = 21 n (%)	Housekeepers N = 25 n (%)	Therapists and Technicians N = 48 n (%)	Others N = 35 n (%)
**Gender**						
Male	125 (61.6)	15 (20.3)	19 (90.5)	25 (100)	37 (77.1)	29 (82.9)
Female	78 (38.4)	59 (79.7) (*p* < 0.0001)	2 (9.5) (*p* < 0.0001)	0	11 (22.9) (*p* < 0.0001)	6 (17.1) (*p* < 0.0001)
**Age**						
<40 years	130 (64)	49 (66.2)	7 (33.3)	15 (60)	34 (70.8)	25 (71.4)
≥40 years	73 (36)	25 (33.8) (*p* = 0.005)	14 (66.7) (*p* = 0.127)	10 (40) (*p* = 0.317)	14 (29.2) (*p* = 0.004)	10 (28.6) (*p* = 0.114)
**Nationality**						
Saudi	75 (36.9)	19 (25.7)	6 (28.6)	0	30 (62.5)	20 (57.1)
Non-Saudi	128 (63.1)	55 (74.3) (*p* < 0.0001)	15 (71.4) (*p* = 0.050)	25 (100)	18 (37.5) (*p* = 0.083)	15 (42.9) (*p* = 0.398)
**Treatment Location**						
Home	169 (83.3)	72 (97.3)	21 (100)	1 (4)	44 (91.7)	31 (88.6)
Hospital	34 (16.7)	2 (2.7) (*p* < 0.0001)	0	24 (96) (*p* < 0.0001)	4 (8.3) (*p* < 0.0001)	4 (11.4) (*p* < 0.0001)

Groups compared using the chi-square (nonparametric) test. *p*-values of <0.05 are considered statistically significant.

## Data Availability

All data are available upon reasonable request to the corresponding author.

## Abbreviations

COVID-19	coronavirus disease 2019
HCWs	healthcare workers
KSA	Kingdom of Saudi Arabia
RT-PCR	real-time reverse transcriptase polymerase chain reaction
